# Mitochondrial Proteins as Exosomal Cargo: New Breast Cancer Biomarkers & Crucial Players in Carcinogenesis?

**DOI:** 10.3390/molecules30204112

**Published:** 2025-10-16

**Authors:** Aleksei Shefer, Lyudmila Yanshole, Alina Grygor’eva, Natalia Yunusova, Irina Kondakova, Liudmila Spirina, Andrey Shevela, Alyona Chernyshova, Alexander Romanov, Svetlana Tamkovich

**Affiliations:** 1 Institute of Chemical Biology and Fundamental Medicine, Siberian Branch of Russian Academy of Sciences, 630090 Novosibirsk, Russia; a.shefer@g.nsu.ru (A.S.); lucy@tomo.nsc.ru (L.Y.); feabelit@mail.ru (A.G.);; 2 Cancer Research Institute, Tomsk National Research Medical Center, Russian Academy of Sciences, 634009 Tomsk, Russia; bochkarevanv@oncology.tomsk.ru (N.Y.);; 3 Department of Biochemistry and Molecular Biology with a Course in Clinical Laboratory Diagnostics, Siberian State Medical University, 634050 Tomsk, Russia; spirinalvl@mail.ru; 4 E.N. Meshalkin National Medical Research Center, Ministry of Health of the Russian Federation, 630055 Novosibirsk, Russia; 5 Cardiovascular Surgery Department, Novosibirsk State Medical University, 630091 Novosibirsk, Russia

**Keywords:** extracellular vesicles, exosomes, mitochondrial proteins, breast cancer, MALDI-TOF mass spectrometry

## Abstract

Mitochondrial proteins (mtPs) packaged into extracellular vesicles (EVs), particularly exosomes, have emerged as promising biomarkers and functional mediators in cancer biology. In this study, we investigated whether breast cancer (BC)-derived exosomes selectively incorporate mtPs, thereby providing insights into their diagnostic potential and role in tumor progression. Exosomes were isolated from conditioned media of multiple BC cell lines and non-tumorigenic breast epithelial cells using ultracentrifugation and were characterized by transmission electron microscopy and flow cytometry for canonical markers CD9 and CD81. Proteomic profiling by MALDI-TOF mass spectrometry revealed a distinct enrichment of mtPs in exosomes derived exclusively from tumor cells, while non-tumorigenic cells lacked such cargo. Identified proteins were predominantly associated with the oxidative phosphorylation system, with Complex I subunits most frequently detected, whereas Complex II components were entirely absent. These findings highlight a cancer-specific mechanism of exosomal mtP incorporation, potentially reflecting altered energy metabolism and stress responses in malignant cells. We conclude that mtPs in exosomes represent both functional contributors to tumor progression and promising candidates for liquid biopsy-based BC diagnostics.

## 1. Introduction

Small extracellular vesicles (sEVs), particularly exosomes, are nanosized particles of approximately 30–150 nm in diameter that mediate intercellular communication by transporting a wide spectrum of signaling molecules. Exosomal molecular cargo includes classical exosomal markers such as TSG101, Alix, CD81 and CD63 and a variety of bioactive molecules: lipids, nucleic acids and proteins. It is noteworthy that exosomal cargo reflects the cell origin, but also represent proofs of directed sorting [1]. Increasing evidence suggests that exosomes may contain mitochondrial proteins (mtPs), mitochondrial DNA (mtDNA) and in some cases even intact mitochondria, which has important implications for disease mechanisms and can be used for diagnostic purposes [2].

One of the earliest demonstrations of mtPs serving as biomarkers within cancer-derived exosomes was reported by Jang et al. [2], who isolated EVs from metastatic melanoma tissues and identified mitochondrial inner membrane proteins such as MT-CO2, COX6c, and the transporter SLC25A22, showing their enrichment relative to healthy controls and their diagnostic potential in multiple tumor types. Similarly, it was demonstrated that therapy-resistant primal breast cancer (BC) cells release exosomes carrying fragments of mtDNA that restore proliferation in dormant cells, thereby supporting resistance to hormonal therapy [3]. Subsequent studies have expanded this concept across other solid tumors. In colorectal carcinoma, another study showed that tumor exosomes reprogram fibroblast metabolism, enhancing oxidative phosphorylation and tricarboxylic acid cycle activity while downregulating mitochondrial translation factors. The affected proteins included ATP5H, IDH2, ECH1, TUFM, and ALDH2, indicating a shift toward an oxidative, “energy-rich” phenotype that supports tumor growth. Elevated mitochondrial chaperones such as HSP60 (HSPD1) in circulating exosomes have also been associated with advanced CRC [4]. In pancreatic ductal adenocarcinoma, patient-derived exosomes were shown to remodel the mitochondrial proteome of recipient cells, leading to increased proliferation, migration, and resistance to gemcitabine. Key proteomic changes included downregulation of TUFM and LRPPRC and upregulation of HADHB, PRDX5, and IMMT, reflecting enhanced fatty acid oxidation, improved ROS detoxification, and remodeling of inner mitochondrial membrane scaffolding [5]. Additional evidence from non-small cell lung cancer, glioblastoma, melanoma, and prostate cancer supports the view that exosomal transport of mitochondrial enzymes—particularly those involved in oxidative phosphorylation and fatty acid oxidation—represents a general mechanism of metabolic adaptation in the tumor microenvironment [6]. Together, these findings indicate that tumor exosomes selectively package mtPs to create metabolic symbiosis within the tumor microenvironment, where stromal cells act as nutrient donors and cancer cells gain enhanced bioenergetics, redox balance, and survival advantages.

Although the strongest body of evidence links mtPs in exosomes to tumor biology, additional studies suggest broader implications. A novel subpopulation of EVs enriched in mtPs, termed mitovesicles, has been described in Down syndrome brains, pointing to a role in neurodegeneration [7]. Mitochondria-containing EVs secreted by immune cells have been shown to induce inflammatory signaling [8], while mitochondria-rich EVs from cardiomyocytes may contribute to restoration of energetic balance in ischemic myocardium [9]. Although less extensively characterized than oncological contexts, these examples underscore the systemic relevance of mitochondrial EVs.

The diagnostic and therapeutic potential of mtPs in EVs is increasingly recognized. MT-CO2 and COX6c in melanoma and other tumors exemplify their role as liquid biopsy biomarkers [2]. Taken together, mtPs in EVs are emerging for studying in sight of diagnostic and prognostic biomarkers so as therapeutic agents, positioning them at the frontier of translational medicine.

In this study, we hypothesized that mtPs are packaged only in tumor-derived exosomes. To test our hypothesis, we selected various cells mimicking normal breast tissue and different subtypes of BC and used mass spectrometry to identify exosomal proteins and search for mtPs in their composition. Bioinformatics analysis confirmed non-random loading of mtPs into cancer exosomes.

## 2. Results

### 2.1. Characterization of Exosomes

**Figure 1:**
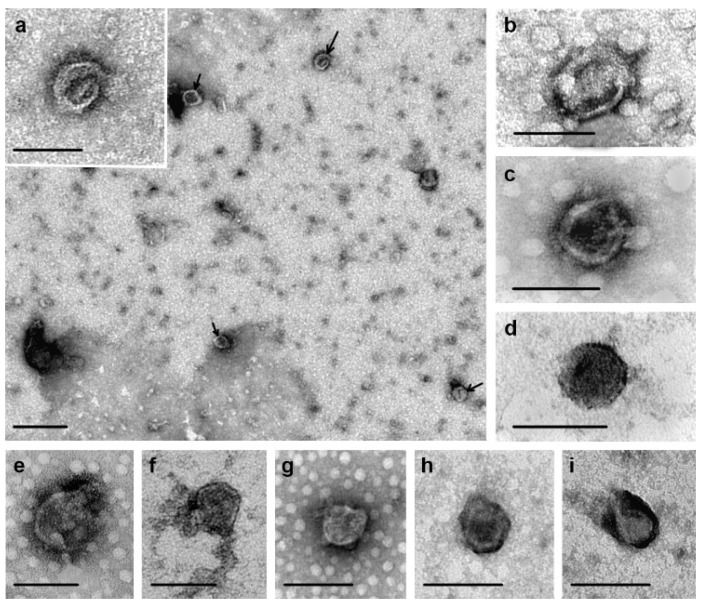
Total view of exosome samples isolated from cell culture medium: (**a**) BT-549, (**b**) BT-474, (**c**) ZR-75-1, (**d**) MDA-MB-231, (**e**) MCF-7, (**f**) SK-BR-3, (**g**) HBL-100, (**h**) T-47D, (**i**) MCF-10A. The length of the scale line corresponds to: (**a**)—200 nm, inset a; (**b**–**i**)—100 nm. Arrows indicate exosomes. Electron microscopy, negative staining by phosphotungstic acid.

Exosomes isolated from conditioned media of breast cell lines were characterized using transmission electron microscopy (TEM) and flow cytometry. TEM analysis revealed homogeneous populations of vesicles exhibiting the typical cup-shaped morphology, with diameters predominantly in the 40–95 nm range, and displaying intact membrane structures (Figure 1). Notably, vesicles smaller than 30 nm accounted for less than 13% of the total particle population.

Flow cytometry profiling further verified the exosomal nature of the isolates, demonstrating consistent expression of the canonical tetraspanin markers CD9 and CD81 across all samples (Table 1, Figure S1).

Together, these results provide robust evidence that the sEVs obtained in this study fulfill the established morphological and molecular criteria of bona fide exosomes.

### 2.2. MtPs in Exosomes Derived from BC Cell Lines

By applying MALDI-TOF mass-spectrometry, we identified the protein constituents of exosomes identified across all examined breast cell lines (Table 2). The proteomic profiles were consistent with classical exosomal markers signatures, thereby confirming the validity of the isolation and characterization procedures (Tables S1–S12).

Importantly, mtPs were exclusively detected in exosomes derived from tumor-mimicking cell lines, whereas they were absent in those secreted by non-tumorigenic epithelial cell lines. This observation suggests a tumor-specific enrichment of mitochondrial components within the exosomal cargo, which may reflect altered bioenergetics states or enhanced mitochondrial turnover characteristic of malignant cells. The overall high heterogeneity of exosomal proteins was evident across different breast cancer subtypes, in line with previous studies, highlighting the complexity and subtype-specific variability of the exosomal proteome [1]. This additionally underscores the importance of exosomes as potential biomarkers for BC subtype identification and stratification.

### 2.3. Exosomal mtPs Are Associated with Cancer Progression

Among the identified mtPs, the majority were functionally associated with components of the oxidative phosphorylation (OXPHOS) system (Table 2). Within this category, proteins belonging to Complex I (NADH:ubiquinone oxidoreductase) were most abundantly represented, indicating a predominant enrichment of this respiratory chain complex in the exosomal proteome. In contrast, no proteins corresponding to Complex II (succinate dehydrogenase complex) were detected across any of the analyzed cell lines. Notably, only one mitochondrial protein not directly linked to the OXPHOS machinery was identified—P36957 (E2 component of the 2-oxoglutarate dehydrogenase complex), which is a key enzyme of the tricarboxylic acid cycle. This distribution suggests that exosomal cargo is selectively enriched in mtPs associated with electron transport and energy metabolism, while non-respiratory mitochondrial components are largely excluded.

Thus, exosomal proteomic profiling demonstrated that mtPs were selectively enriched in tumor-derived cell lines, with most of them associated with the oxidative phosphorylation system, particularly Complex I. The absence of Complex II proteins and the rare detection of non-OXPHOS proteins, such as DLST (P36957), suggest a specific pattern of mitochondrial protein incorporation into exosomes linked to cancer-related metabolic processes.

**Figure 2:**
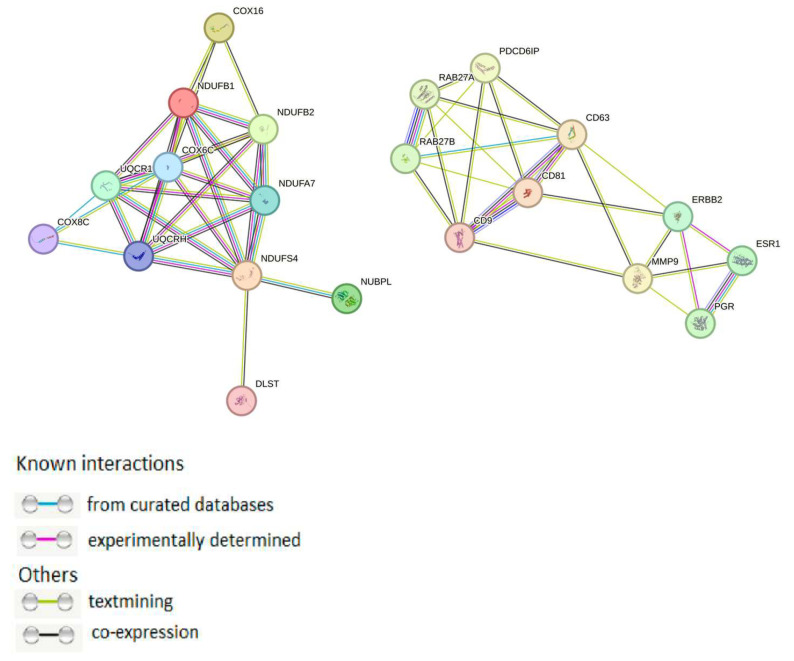
PPI network of mtPs in exosomes and major proteins implicated in exosomal sorting. PPI networks plotted with the STRING (http://string-db.org/, accessed on 10 September 2025) with the following settings—minimum interaction score: confidence: 0.400; active interaction sources: textmining, experiments, databases, co-expression, neighborhood, gene fusion, co-occurrence.

Using the STRING web tool, protein–protein interactions (PPIs) network for mtPs and proteins most strongly involved in exosome sorting, as well as with cancer-associated receptors and major exosomal proteins (ALIX, Rab-proteins, tetraspanins and matrix metalloproteases (MMPs) was constructed (Figure 2). It was found that none of the mtPs identified in our study interacted directly with those proteins.

**Figure 3:**
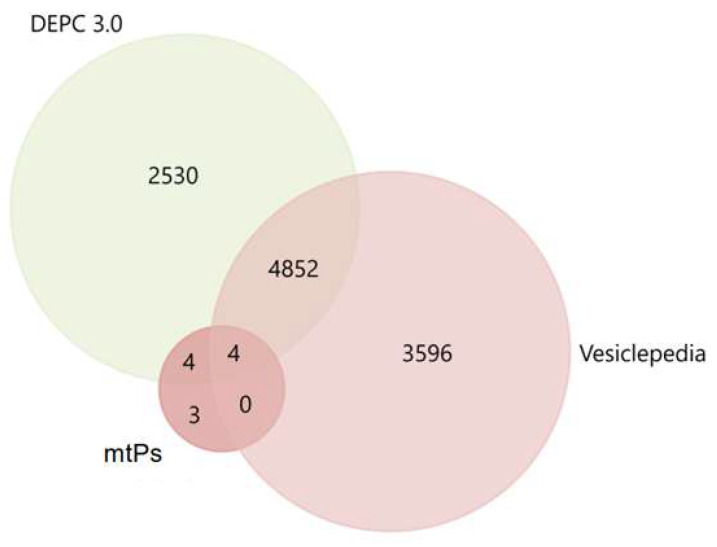
The Venn–Euler diagram of mtPs in dbDEPC and dbVesiclepedia, composed using the QuickGO 2025-06-01 and FunRich 3.13 software.

For a primary evaluation of the potential involvement of the mitochondrial proteins identified in our dataset in vesicular trafficking and tumor-associated processes, we performed enrichment analysis using FunRich 3.1.3 with reference to Vesiclepedia and DEPC 3.0 databases. The cross-comparison with Vesiclepedia revealed that only four of the proteins detected in our study (*DLST*, *NDUFS4*, *COX6C*, *UQCR10*) had previously been reported as components of EVs (Figure 3).

This relatively small overlap highlights the novelty of our dataset and suggests that a substantial fraction of mitochondrial proteins recruited into exosomes in BC cells may represent previously unrecognized cargo species. Importantly, however, the interrogation of the DEPC 3.0 database showed that nearly all of the identified proteins, with the exception of only three (*COX16*, *NDUFB2*, *COX8C*), are consistently annotated as being altered during tumor dissemination and metastatic progression. Such concordance strongly reinforces the idea that mitochondrial proteins are not passive bystanders in EVs, but are instead actively engaged in tumor-associated processes.

Taken together, these observations underscore the dual significance of our findings: on the one hand, mitochondrial proteins appear to represent a highly relevant class of tumor-associated exosomal cargo, while on the other hand, vesicle-mediated transport itself emerges as a key pathway through which malignant cells may exploit mitochondrial components to support cancer progression.

## 3. Discussion

The significance of mtPs in cargo of exosomes was shown by several independent studies. For example, MT-CO2 and COX6c, which was observed in cargo of highly aggressive triple-negative BC cell line BT-549 were demonstrated to be significantly elevated in melanoma-derived EVs and can also be detected in plasma of breast and ovarian cancer patients, highlighting their diagnostic potential [2]. Similarly, it was reported that BC-derived EVs enriched in mitochondrial respiratory proteins enhance oxidative metabolism and ATP production in recipient cells, indicating that such vesicles can actively reprogram the tumor microenvironment [10]. In addition, Shafiq et al. [11] profiled microvesicles from epithelial cells undergoing H-Ras-induced epithelial–mesenchymal transition (EMT) and found that the post-EMT vesicles were uniquely enriched in mtPs. Functionally, these vesicles enhanced migration, anchorage-independent growth, and EMT in recipient epithelial cells, demonstrating that mtPs delivered by EVs can directly promote metastatic behavior. Complementing these findings, another study showed that EVs from highly metastatic lung cancer cells induced a cancer-associated fibroblast phenotype in normal fibroblasts, characterized by elevated mitophagy and ROS production. These fibroblasts released abundant mtDNA, which was subsequently taken up by nearby cancer cells lacking mitochondrial genomes [12]. The transferred mtDNA restored mitochondrial respiration and OXPHOS in recipient cells and increased their resistance to oxidative stress, thus illustrating how EV-mediated mitochondrial exchange can support proliferation and metastasis under stress conditions. Taken together, these results support the idea that mtPs in exosomes are not only functional agents of tumor progression but can also be used as markers in cancer diagnostics.

Our results on the identification of mtPs in exosomes derived from breast cell lines highlight at least three important issues. First, the selective enrichment of mtPs in exosomes from tumor-derived, but not non-tumorigenic, breast epithelial cells suggest a cancer-specific mechanism of cargo loading. This tumor-dependent enrichment points to a potential role of mitochondrial components in malignant cell communication and tumor progression. Second, most of the identified mtPs belonged to the oxidative phosphorylation machinery, in particular to Complex I, whereas proteins of Complex II were entirely absent. Such a selective profile strongly indicates that oxidative metabolism and mitochondrial stress states may guide the recruitment of mtPs into exosomes. It is noteworthy that succinate dehydrogenase is the only OXPHOS complex encoded exclusively by nuclear DNA rather than the mitochondrial genome [13], which further underscores the specificity of mtPs sorting into exosomes. Third, the heterogeneity in the number and type of mtPs detected across different BC subtypes suggests that hormonal or metabolic states of the tumor may influence this process, though the precise mechanisms remain unknown.

The mechanisms underlying the selective incorporation of mtPs into exosomes are currently poorly understood. Available data point to a link with mitochondrial oxidative stress and quality control pathways. For example, it has been demonstrated that functional mtPs are selectively package into exosomes under homeostatic conditions, while damaged proteins are degraded via Parkin-dependent mitophagy [14]. At the same time, data from other studies suggest that reactive oxygen species (ROS) and mitochondrial dysfunction may be key triggers for the sorting of mitochondrial proteins and mtDNA into exosomes [12]. Bioinformatics analysis revealed that none of the proteins identified in our study interacted directly with those proteins most strongly involved in exosome sorting, as well as with BC-associated receptors. However, it is also known that inhibition of these sorting-related proteins only reduces the amount of secreted exosomes by approximately 50%, indicating the existence of an alternative pathway for protein incorporation into exosomes [15]. These observations suggest that the interplay between oxidative stress, mitochondrial quality control, and vesicular trafficking defines the composition of exosomal cargo.

Finally, we have searched if the proteins matched in DEPC 3.0 were mentioned in other publications as potential biomarkers. In particular *NDUFB1*—an accessory, non-catalytic subunit of mitochondrial Complex I, which contributes to the assembly and stabilization of Complex I. Dysregulation of Complex I subunits, including *NDUFB1*, is frequently observed in cancer, reflecting metabolic rewiring of malignant cells. For instance, *NDUFB1* mRNA was reported to be downregulated in aggressive ovarian cancers, suggesting a reduction in oxidative phosphorylation in high-risk tumors [16], whereas altered transcript levels were also detected in clear-cell renal cell carcinoma [17]. In general, perturbations of Complex I function, including those involving *NDUFB1*, affect the NAD^+^/NADH balance, modulate reactive oxygen species production, and contribute to the metabolic switch characteristic of tumor cells [18]. Although *NDUFB1* has not been validated as an independent biomarker, it has appeared in multi-gene prognostic signatures, such as a poor-prognosis ovarian cancer panel incorporating low *NDUFB1* expression [17].

Another Complex I subunit, *NDUFS4*, also known as NADH ubiquinone oxidoreductase subunit S4, is an iron–sulfur protein essential for Complex I assembly and enzymatic activity. In cancer, *NDUFS4* expression has been linked to tumor progression: in gastric cancer, it is significantly overexpressed compared to normal tissue, with elevated levels correlating with advanced stage and poor survival [19]. Experimental silencing of *NDUFS4* reduced proliferation and invasion of gastric cancer cells, while in xenograft models tumor growth was markedly suppressed [20]. Interestingly, recent findings indicate that loss of *NDUFS4* can also influence tumor immunity. A 2025 Nature Cancer study demonstrated that deletion of *NDUFS4* in mouse models of melanoma and BC increased mitochondrial acetyl-CoA production, enhanced MHC-I antigen presentation, and sensitized tumors to T-cell-mediated cytotoxicity and checkpoint blockade [20]. These results underscore the dual role of *NDUFS4* in promoting tumor metabolism and facilitating immune evasion.

In addition to catalytic subunits, assembly factors also contribute to the functional integrity of Complex I, *NUBPL* (also referred to as IND1 or huInd1) is a mitochondrial assembly protein responsible for incorporating iron–sulfur clusters into Complex I. Aberrant *NUBPL* expression has been associated with cancer progression, particularly in colorectal carcinoma, where it is significantly upregulated in tumors, especially in metastatic lesions. Overexpression of *NUBPL* enhanced migration and invasion in colorectal cancer cells, while silencing suppressed these malignant traits [21]. Mechanistically, *NUBPL* activated ERK-signaling and promoted EMT, as reflected by downregulation of E-cadherin and upregulation of mesenchymal markers [21]. Further studies have implicated *NUBPL* dysregulation in gastric cancer and bladder cancer, with high *NUBPL* expression predicting poor prognosis and reduced responsiveness to immune checkpoint inhibitors [22]. These findings support the notion of *NUBPL* as a metastasis-related gene and a potential prognostic biomarker, though clinical validation is still pending.

Other mitochondrial respiratory chain subunits identified in our dataset exhibit more limited evidence of cancer involvement. *UQCR10*, a small subunit of Complex III, contributes to the assembly of the Rieske iron–sulfur protein, but has not been consistently linked to tumorigenesis. The only reported cancer-related alteration is a rare *UQCR10-C1ORF194* fusion detected in spinal ependymoma [23], with no clear functional role attributed to this event.

Similarly, *NDUFA7*, an accessory subunit of Complex I, has not been directly implicated in tumor biology. Although minor expression changes have been reported in gastric cancer datasets [24], no functional or prognostic studies are available, and its role in carcinogenesis remains essentially unexplored.

By contrast, *COX6C*, a nuclear-encoded subunit of cytochrome c oxidase (Complex IV), is frequently dysregulated in a variety of malignancies. Overexpression of *COX6C* has been reported in breast, prostate, thyroid, gastric cancers, melanoma, and uterine leiomyoma [25]. Functionally, elevated *COX6C* expression enhances oxidative phosphorylation and supports tumor proliferation, as demonstrated in gastric and prostate cancer cells [26,27]. Furthermore, *COX6C* participates in oncogenic rearrangements, including gene fusions with *HMGA2* in uterine leiomyoma and translocations in retroperitoneal lipomas and thyroid carcinomas [28,29]. Notably, circulating *COX6C*-containing vesicles have been proposed as potential biomarkers for melanoma [25,29]. Collectively, these observations suggest that *COX6C* contributes to tumor metabolism and may hold promise as a prognostic biomarker, though further clinical validation is required.

Another subunit of Complex III, *UQCRH*, also displays context-dependent roles in cancer. In clear-cell renal cell carcinoma, *UQCRH* is frequently silenced by promoter hypermethylation, with low expression correlating with advanced disease and poor survival, suggesting a tumor-suppressive role [30]. Conversely, in hepatocellular carcinoma, *UQCRH* is commonly overexpressed and correlates with tumor size, vascular invasion, poor differentiation, and shorter patient survival, making it an adverse prognostic factor [30]. Thus, *UQCRH* appears to act as either a tumor suppressor or promoter depending on tissue context, yet in both cases, its expression levels serve as clinically relevant prognostic indicators.

Finally, *DLST*, the E2 component of the α-ketoglutarate dehydrogenase complex, has recently been linked to tumor aggressiveness. Elevated *DLST* expression marks highly aggressive, treatment-resistant neuroblastomas, where it predicts poor outcome [31]. This functional study in zebrafish and mammalian models revealed that *DLST* promotes tumor progression by fueling oxidative phosphorylation, whereas its depletion induces apoptosis. In triple-negative BC, *DLST* overexpression correlates with significantly worse overall and relapse-free survival [32], and silencing *DLST* suppresses tumor growth in subsets of BC cells reliant on glutamine-driven TCA cycle metabolism. These findings position *DLST* as both a prognostic biomarker and a potential therapeutic vulnerability in tumors with high oxidative metabolic dependence.

To verify this data, we decided to analyze possible involvement of whole complexes in BC dissemination. For example, multiple Complex I subunits, such as *NDUFB1*, *NDUFS4*, *NUBPL*, and *NDUFA7*, have been implicated in the metabolic plasticity of breast cancer cells. Elevated *NDUFB1* expression correlated with improved relapse-free survival in breast cancer, while its regulation by TNF-α differed between ER-positive and triple-negative cell lines [33]. In contrast, genetic ablation of *NDUFS4* enhanced tumor immunogenicity and restricted tumor growth in vivo, underscoring a role for Complex I in modulating antitumor immunity [20]. Similarly, the Fe-S cluster transfer protein *NUBPL* has been shown to promote epithelial–mesenchymal transition and metastasis in malignant cells [34], and *NDUFA7* expression is repressed by tumor suppressor Prox1, linking this subunit to the regulation of glycolytic metabolism [35]. Complex III components also appear relevant. *UQCRH* loss was associated with promoter hypermethylation in breast and ovarian cancers and promoted a glycolytic switch, while re-expression restored mitochondrial respiration [36]. Although *UQCR10* has been detected in breast tumors, experimental studies did not consistently support a prognostic role [36]. For Complex IV, the assembly factor *COX16* was recently found in a fusion transcript (SYNJ2BP–*COX16*) that drives mitochondrial fragmentation, EMT, and metastatic progression in breast cancer cells [37]. *COX6C*, another subunit of Complex IV, supports drug resistance in ER-positive cell lines by sustaining ATP production required for ABCG2 efflux activity, and was also included in multi-gene survival prediction models [38]. By contrast, *COX8C* has not yet been functionally linked to breast cancer pathogenesis.

Finally, mitochondrial metabolism beyond the respiratory chain contributes as well. *DLST*, the E2 component of the 2-oxoglutarate dehydrogenase complex, was identified as a dependency in subsets of triple-negative breast cancer, where its high expression was associated with poor prognosis. Functional suppression of *DLST* impaired growth and increased oxidative stress in *TNBC* models, highlighting its potential as a therapeutic target [32].

Taken together, the evidence summarized above demonstrates that multiple mtPs, including structural subunits and assembly factors of the respiratory chain as well as key metabolic enzymes, display cancer type-specific patterns of dysregulation. Their overexpression, downregulation, or context-dependent alterations are consistently associated with tumor progression, metabolic adaptation, metastatic potential, and, in some cases, patient prognosis. The fact that these proteins are selectively incorporated into exosomes derived from malignant cells further emphasizes their functional relevance in intercellular communication within the tumor microenvironment. Collectively, these observations strongly support the notion that changes in the expression of mitochondrial proteins in exosomes can indeed serve as informative markers of tumor-associated processes and may provide a valuable basis for the development of prognostic or diagnostic approaches in oncology.

Nevertheless, several limitations must be considered when interpreting these findings. First, the associations between specific mitochondrial proteins and breast cancer pathogenesis are derived from cell line models, which may not fully capture the heterogeneity of the disease. Second, the functional contribution of individual subunits or assembly factors is often inferred indirectly, as their dysregulation may reflect global alterations of mitochondrial complexes rather than a direct causal role. Third, technical challenges such as variability in exosome isolation, incomplete separation of tumor-derived vesicles from other extracellular particles, and biases introduced during sample processing may affect the reproducibility and comparability of proteomic data across studies. Finally, the integration of MALDI-TOF MS results with other proteomic and genomic platforms is still needed to improve sensitivity and specificity of biomarker discovery in clinical settings. Despite these limitations, the present work contributes to the growing understanding of mitochondrial proteins in breast cancer biology and highlights their potential as promising biomarkers and therapeutic targets in oncology research.

## 4. Materials and Methods

### 4.1. Cell Lines

Cell lines applied in this study were obtained from American Type Culture Collection (ATCC) including SK-BR-3 (HER2-positive BC cells), (Catalog # HTB-30, ATCC, USA); BT-474 (triple-positive BC cells), (Catalog # HTB-20, ATCC, USA); ZR-75-1 (hormone receptor-positive BC cells), (Catalog # CRL-1500, ATCC, USA); MCF-7 (hormone receptor-positive BC cells), (Catalog # HTB-22, ATCC, USA); T-47D (hormone receptor-positive BC cells), (Catalog # HTB-133, ATCC, USA); BT-549 (triple-negative BC cells), (Catalog # HTB-122, ATCC, USA); HCC-1937 (triple-negative BC cells), (Catalog # CRL-2336, ATCC, USA); MDA-MB-231 (triple-negative BC cells), (Catalog # HTB-26, ATCC, USA); MDA-MB-463 (triple-negative BC cells), (Catalog # HTB-132, ATCC, USA); HBL-100 (non-tumorigenic breast epithelial cells), (Catalog # CRL-2321, ATCC, USA). These cells were cultured in Dulbecco’s Modified Eagle’s Medium (DMEM) supplemented with 10% Fetal Bovine Serum (FBS) (Thermo Fisher Scientific, Waltham, MA, USA) and Penicillin-Streptomycin (100 μg/mL) (Thermo Fisher Scientific, Waltham, MA, USA) in a CO_2_-incubator (5% 335 CO_2_) at 37 °C up to 70–80% confluence.

The non-tumorigenic human breast epithelial cell line MCF-10A (Catalog #CRL-10317, ATCC, USA) was cultured in DMEM/F12 medium supplemented with 5% Horse Serum (Gibco, New Zealand), 20 ng/mL Epidermal Growth Factor (EGF), 0.5 μg/mL Hydrocortisone, 10 μg/mL Insulin, and 100 ng/mL Cholera Toxin (Sigma, St. Louis, MO, USA). Cells were maintained in a CO_2_-incubator (5% CO_2_) at 37 °C until they reached 70–80% confluence.

Prior to use, FBS and Horse Serum were centrifuged at 100,000 g for 2 h at 4 °C to remove EVs. The supernatant was collected and used to prepare EV-depleted medium. Three days before cell harvesting, the culture medium was replaced with depleted medium containing all other ingredients, described previously.

### 4.2. Exosomes Isolation

After 48 h of cultivation of cell lines, conditioned medium was collected, and cells were pelleted by centrifugation at ambient temperature for 5 min at 800 g. The supernatant was then centrifuged at 4 °C for 20 min at 15,000 g to remove cellular debris. To eliminate large EVs, the supernatant was filtered through 100 nm pore-size filters (Minisart high flow, 16553-K, Sartorius, Goettingen, Germany).

The filtrate was centrifuged for 90 min at 100,000 g at 4 °C. The pellets were suspended in 12 mL of PBS and centrifuged again for 90 min at 100,000 g at 4 °C. This washing step was repeated twice. Afterwards, the supernatant was removed, and pellets were resuspended in 300 μL of PBS, aliquoted, frozen in liquid nitrogen, and stored at –80 °C.

### 4.3. TEM

Morphology and membrane integrity of exosomes were assessed by TEM as described previously [39]. Specifically, 15 μL of exosomes were sorbed onto a copper grid coated with carbon-stabilized film, followed by addition of 2% phosphotungstic acid. Exosomes were examined on a TEM (JEM 1400, Jeol, Tokyo, Japan) equipped with a Veleta digital camera (Olympus Corporation, Tokyo, Japan). Vesicle sizes were estimated using iTEM software ver. 2.5 (Olympus Corporation, Tokyo, Japan).

### 4.4. Flow Cytometry

For flow cytometric analysis, aldehyde/sulfate latex beads (4 μm diameter; Invitrogen, Carlsbad, CA, USA) were incubated with purified anti-CD9 antibodies (BD Biosciences, San Jose, CA, USA) at 22 °C overnight with gentle agitation, as previously described [39]. Subsequently, 30 µg of exosomes were incubated with 3 × 10^5^ antibody-coated beads in PBS at 4 °C overnight. The reaction was quenched with 0.2 M glycine for 30 min and bead–exosome complexes were washed twice in FACS buffer (3% exosome-depleted FBS in PBS). Complexes were then incubated with fluorescein-conjugated anti-CD81 or isotype control antibodies (BD Biosciences, San Jose, CA, USA) for 40 min at room temperature, washed, and resuspended in 300 µL of FACS buffer. Flow cytometry was performed on the Cytoflex (Beckman Coulter, BioBay, Suzhou, China) using CytExpert 2.0 Software.

### 4.5. Mass Spectrometry Analysis

The amount of exosomes used for MALDI-TOF identification was normalized to protein equivalents using the NanoOrange^®^ Protein Quantitation Kit (Molecular Probes, Eugene, OR, USA), following the manufacturer’s instructions. For the identification of exosomal proteins by MALDI-TOF mass spectrometry, proteins from cell lines derived exosomes were first separated with SDS-PAGE with 4.5% stacking gel and 10% resolving gel. Gel lanes were sliced into 0.5 cm strips up to 23 cm and placed into 1.5 mL Eppendorf tubes. Gel pieces were washed twice with 200 μL of 0.2 M (NH_4_)_2_CO_3_ and 50% acetonitrile at 37 °C for 30 min with shaking. The solution was removed under vacuum, and gel pieces were dried with 100 μL of 100% acetonitrile for 10 min.

After drying, 0.2 mM trypsin solution (T6567, Sigma, USA) in 40 mM (NH_4_)_2_CO_3_ and 5 μM CaCl_2_ was added and incubated for 30 min at room temperature. Then, 60 μL of peptide extraction buffer (40 mM (NH_4_)_2_CO_3_, 5 μM CaCl_2_, 10% acetonitrile) was added, and samples were incubated with shacking for 12 h at 37 °C. Before MALDI-TOF analysis, peptides were desalted using C-18 ZipTip columns (Millipore, Darmstadt, Germany). Columns were washed with 100% acetonitrile, 50% acetonitrile, and 0.1% trifluoroacetic acid. Peptides were eluted with α-Cyano-4-Hydroxycinnamic Acid (HCCA) in 70% acetonitrile and spotted onto MTP 384 target plates. After crystallization, spectra were acquired with standard calibration (mass range 500–3800 kDa).

Protein identification was performed using BioTools ver. 3.2. and Mascot Daemon ver. 3.0 software by searching the SwissProt database (Homo sapiens, tolerance ±300 ppm, ≤2 missed cleavages, fixed modification – Propionamide (C), variable modifications – Oxidation (M), Phospho (ST)). Only identifications with ≥95% reliability and ≥2 peptides matched were considered valid identification reliability not lower than 95%. Mass-spectra were registered at the Centre of collective usage “Mass-spectrometric studies” of Siberian Branch of Russian Academy of Science.

Coverage of about 10–20% of the protein sequence, with at least two peptides matching, was considered as reliable identification of minor proteins [40,41,42].

### 4.6. Bioinformatics Analysis

Data obtained from exosomal proteomes were mapped in the UniProt database (https://www.uniprot.org accessed on 24 May 2025) using the Retrieve/ID mapping platform. Functional enrichment analysis was carried out with the STRING platform (https://www.string-db.org accessed on 3 June 2025). Involvement in tumor-associated processes was investigated with the use of the DEPC 3.0 database [43], and involvement in EV-associated transport was analyzed with the use of the Vesiclepedia database (http://www.microvesicles.org/ (accessed on 12 June 2025).

## 5. Conclusions

Taken together, our results and the literature suggest that mtPs are not randomly recruited into exosomes and may be useful as diagnostic biomarkers in malignancies, including BC. However, the unanswered question of how exactly these proteins are recruited into exosomes, whether via oxidative stress-driven MDV pathways, mitophagy, or other mechanisms, represents a cutting-edge area of translational research. Elucidating these processes will have important implications for understanding tumor metabolism and stress adaptation.

## Figures and Tables

**Table 1:** Expression of CD9 and CD81 on the surface of exosomes from culture medium.

BC Subtype	Cell Line	MFI ^1^ CD9+ CD81+
Non-tumorogenic breast cell lines	HBL-100	1750
	MCF-10A	4050
Luminal A BC cell lines	MCF-7	3170
	ZR-75-1	2120
	T-47D	4120
Triple-positive BC cell line	BT-474	2870
HER2+ BC cell lines	SK-BR-3	2330
Triple-negative BC cell lines	BT-549	4800
	MDA-MB-231	4075
	HCC-1937	1630
Negative control		520

^1^ MFI—the median fluorescence intensity.

**Table 2:** Mass spectrometric identification of major MTPs in exosomes secreted by BC cell lines.

BC Subtype	Cell Line	Gene	Uniprot ID	Protein Name	Mitochondria Component
HER2+	SKBR-3	*NDUFB1*	O75438	NADH dehydrogenase [ubiquinone] 1 beta subcomplex subunit 1	Complex I
Triplepositive	BT-474	*NDUFB1*	O75438	NADH dehydrogenase [ubiquinone] 1 beta subcomplex subunit 1	Complex I
		*NDUFS4*	O43181	NADH dehydrogenase [ubiquinone] iron-sulfur protein 4	Complex I
		*COX16*	Q9P0S2	Cytochrome c oxidase assembly protein COX16 homolog	Complex IV
Luminal A	ZR-75-1	*NDUFB2*	O95178	NADH dehydrogenase [ubiquinone] 1 beta subcomplex subunit 2	Complex I
	MCF-7	*NUBPL*	Q8TB37	Iron-sulfur cluster transfer protein *NUBPL*	Complex I
	T-47D	*UQCR10*	Q9UDW1	Cytochrome b-c1 complex subunit 9	Complex III
Triplenegative	BT-549	*NDUFA7*	O95182	NADH dehydrogenase [ubiquinone] 1 alpha subcomplex subunit 7	Complex I
		*COX6C*	P09669	Cytochrome c oxidase subunit 6C	Complex IV
	HCC-1937	*UQCRH*	P07919	Cytochrome b-c1 complex subunit 6	Complex III
	MDA-MD-463	*COX8C*	Q7Z4L0	Cytochrome c oxidase subunit 8C	Complex IV
	MDA-MB-231	*DLST*	P36957	Dihy-drolipoyllysine-residue succinyltransferase component of 2-oxoglutarate dehy-drogenase complex	Tricarboxylic acid cycle component

## Data Availability

The data presented in this study are available on request from the corresponding author.

## Supplementary Materials

The following supporting information can be downloaded at: https://www.mdpi.com/article/10.3390/molecules30204112/s1, Figure S1: CD81 expression on CD9-positive exosomes from BC cell cultures and non-tumorigenic human breast epithelial cells; Tables S1–S12: Mass-spectrometry identification of major exosomal proteins and mtPs secreted by breast cancer cell lines in exosomes.

## Abbreviations

The following abbreviations are used in this manuscript:

BC	breast cancer
EMT	epithelial–mesenchymal transition
EVs	extracellular vesicles
mtDNA	mitochondrial DNA
mtPs	mitochondrial proteins
OXPHOS	oxidative phosphorylation
PPI	protein–protein interactions
TEM	transmission electron microscopy
