# Supplementary figures and images for: Mitochondrial Proteins as Exosomal Cargo: New Breast Cancer Biomarkers & Crucial Players in Carcinogenesis?
Source: Molecules. 2025 Oct 16;30(20):4112. doi: 10.3390/molecules30204112 (PMC12565999; doi:10.3390/molecules30204112)

## Slide 1
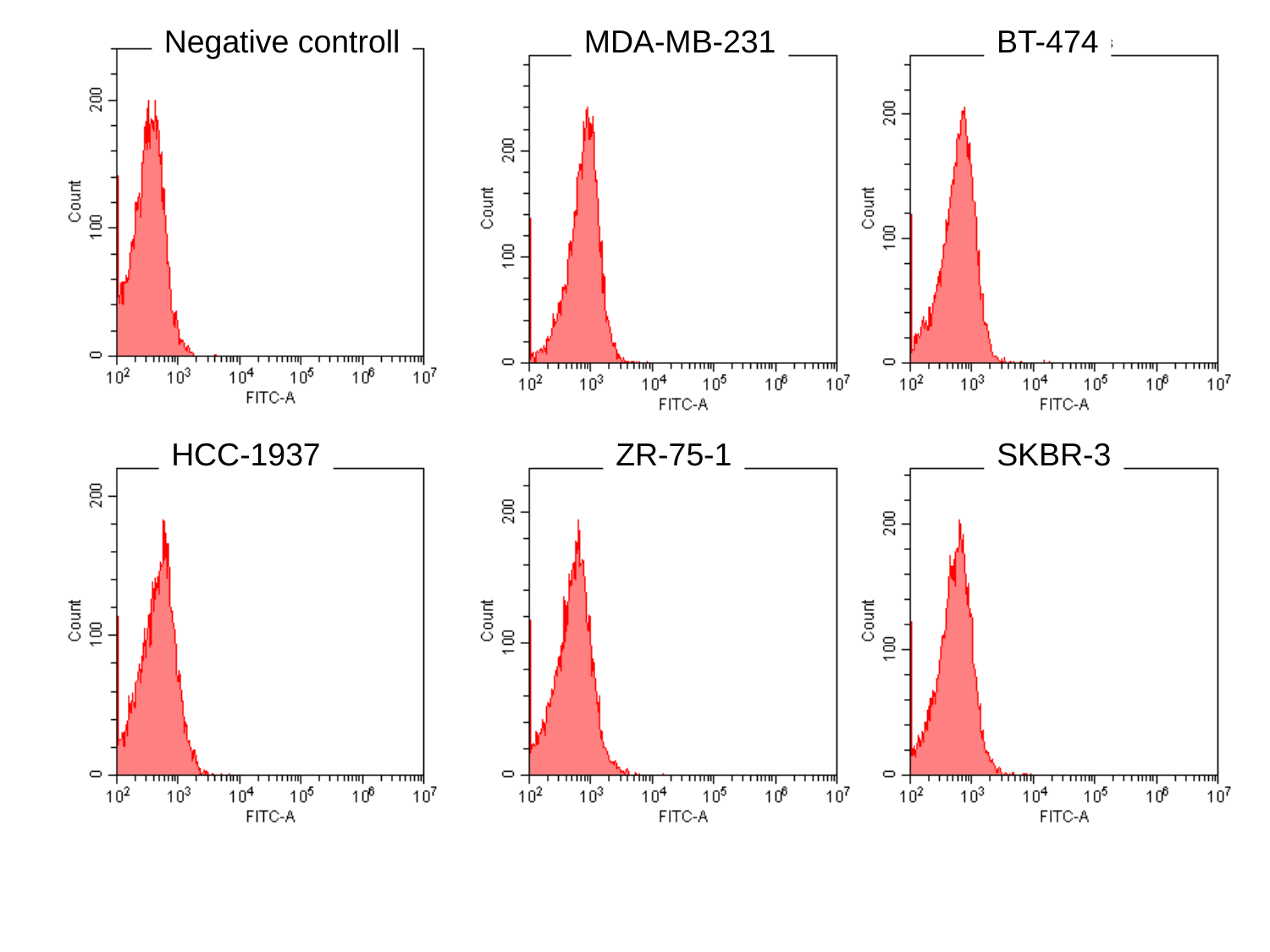

Negative controll
MDA-MB-231
BT-474
HCC-1937
ZR-75-1
SKBR-3

## Slide 2
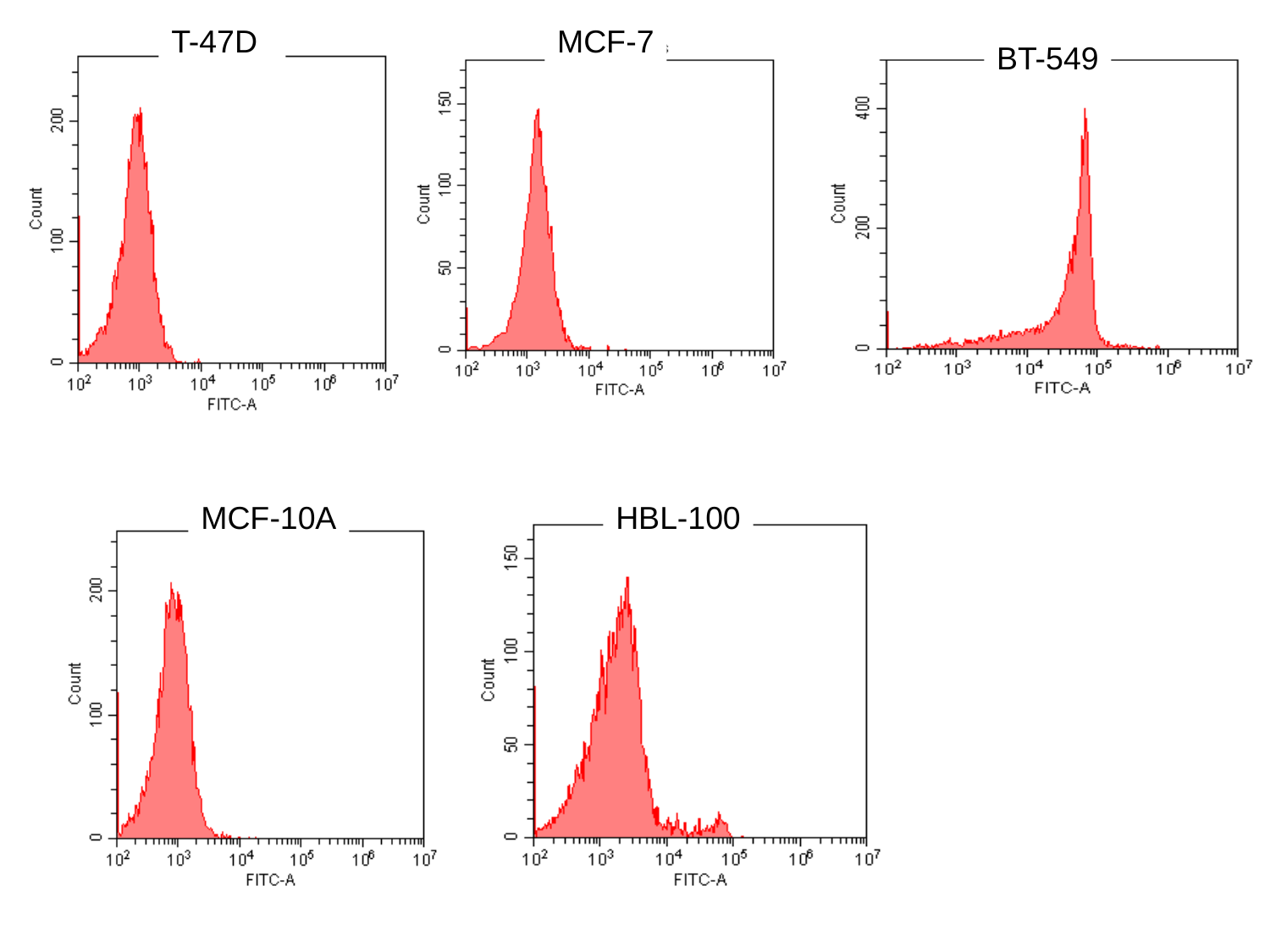

T-47D
MCF-7
BT-549
HBL-100
MCF-10A

Supplement: Supplementary file 1 [file molecules-30-04112-s001.zip › Suppl Fig 1.pptx]
